# Flaxseed and Camelina Meals as Potential Sources of Health-Beneficial Compounds

**DOI:** 10.3390/plants10010156

**Published:** 2021-01-14

**Authors:** Silvia Tavarini, Marinella De Leo, Roberto Matteo, Luca Lazzeri, Alessandra Braca, Luciana G. Angelini

**Affiliations:** 1 Department of Agricultural, Food and Environment, University of Pisa, 56124 Pisa, Italy; silvia.tavarini@unipi.it (S.T.); luciana.angelini@unipi.it (L.G.A.); 2 Research Centre for Nutraceutical and Healthy Foods “NUTRAFOOD”, University of Pisa, 56124 Pisa, Italy; marinella.deleo@unipi.it; 3 Department of Pharmacy, University of Pisa, 56126 Pisa, Italy; 4 Council for Agricultural Research and Economics (CREA), Research Centre for Cereal and Industrial Crops, 40129 Bologna, Italy; roberto.matteo@crea.gov.it (R.M.); luca.lazzeri@crea.gov.it (L.L.)

**Keywords:** *Linum usitatissimum*, *Camelina sativa*, antioxidant capacity, bioactive compounds, glucosinolates, lignans, phenols, co-products valorization

## Abstract

Seed meals and cakes, deriving from minor oilseed crops, represent interesting co-products for the presence of a high content of proteins and bioactive compounds that could be successfully explored as valuable plant-derived feedstocks for food and non-food purposes. In this contest, flaxseed (*Linum usitatissimum* L.) and camelina (*Camelina sativa* (L.) Crantz) are becoming increasingly important in the health food market as functional foods and cosmetic ingredients. Thus, this study aimed to evaluate the effect of genetic characteristics and cultivation sites on the chemical features of seed meals deriving from two flaxseed varieties (Sideral and Buenos Aires) and a camelina cultivar (Italia), cultivated in Central and Northern Italy (Pisa and Bologna). The content of total phenols and flavonoids, seed oil, proteins and fatty acids have been evaluated, together with the chemical profiles of flaxseed and camelina meals. In addition, radical-scavenging activity has been investigated. All the examined seed meals resulted as rich in bioactive compounds. In particular, flaxseed meal is a good source of the lignan secoisolariciresinol diglucoside (SDG) and hydroxycinnamic acid glucosides, while camelina meal contains glucosinolates and quercetin glycosides. Furthermore, all extracts exhibited a very strong radical-scavenging activity, that make these plant-derived products interesting sources for food or cosmetic ingredients with health outcomes.

## 1. Introduction

In recent years, new perspectives for oilseed crops have revealed them to be renewable and valuable feedstocks for biorefinery processes, responding to the urgent need to transition toward a circular economy model based on the zero-waste concept [1]. These crops, in fact, are particularly suitable for obtaining, through a cascading use of total biomass, highest added-value products (pharmaceuticals, nutraceuticals, fine chemicals, cosmetics, agrochemicals, biomaterials), over bioenergy. In particular, oilseed meals and cakes, deriving from seed oil extraction, represent interesting co-products due to their high protein content, also for the presence of bioactive substances, such as phenolic acids, flavonoids, lignans and other antioxidant compounds [2], which could be used as food additives, supplements or cosmeceutical additives for foods and human health protection. In addition to the main oilseed crops, such as soybean and rapeseed used for food, feed and biofuel production, there is a growing interest in other minor oilseed crops suitable for marginal land, which could have a positive impact on the sustainability and resilience of agroecosystems. In this contest, flaxseed (*Linum usitatissimum* L., family Linaceae) and camelina (*Camelina sativa* (L.) Crantz, family Brassicaceae) are becoming more and more important in the health food market as a functional food and cosmetic ingredients [3,4]. Both crops, compared to the traditional oilseeds, display several agronomic advantages, such as great adaptability and phenotypic plasticity, low water and nutrient requirements, as well as good tolerance to pests and pathogens [5,6]. These positive agronomic attributes make these oilseed crops promising to be introduced in Mediterranean agroecosystems, where they might represent useful tools for enhancing biodiversity and cropping system diversification. These crops are not only characterized by positive agronomic traits, but they also have interesting chemical and functional features due to their products and co-products compositions. The nutritional importance of flaxseed is due to the high content of proteins (22%), lipids (43%) and minerals (3%). Its oil represents an important source of omega-3 fatty acids, especially linolenic acid (ALA) (more than 50% of the total fatty acids) [7]. Furthermore, flaxseed seeds, oil and cake are the richest source of the lignan secoisolariciresinol diglucoside (SDG), a natural cancer chemopreventive agent [8], in form of high molecular oligomers. At the same time, the high potential of camelina for nutritional applications is attributed to the distinctive fatty acid composition of its oil, rich in alpha-linolenic (18:3) and linoleic (18:2) acids [9,10]. Being an essential omega-3, alpha-linolenic acid has beneficial health effects on humans [11]. The presence of eicosenoic acid (11–19%) and tocopherols in relatively large amounts, and the low content of anti-nutritionals such as erucic acid, are additional distinctive differences of camelina in comparison with other commonly used vegetable oils [9,12]. All these compounds have antioxidant and free-radical scavenging activities and can play an important role in preventing several human diseases thanks their potential anti-tumoral, antiviral, antibacterial, and anti-mutagenic abilities [13]. For the aforementioned properties, flaxseed and camelina meals have interesting usable potential as ingredients for food and non-food purposes. Cake/meals composition is known to have a wide range of variability, depending on genetic and environmental growing conditions, and the extraction method. However, the current state of knowledge about the chemical composition of camelina and flaxseed meals, depending on the variety/cultivar and environmental conditions in which the plant is grown, is scarce. Therefore, the present study aimed to evaluate the role of environment and genotype in defining the chemical features of flaxseed and camelina meals. Consequently, the seed meals, obtained after solvent oil extraction, deriving from two flaxseed varieties (Sideral and Buenos Aires) and a camelina cultivar (Italia), cultivated in two environments of central and northern Italy (Pisa and Bologna), were analyzed for their phytochemical content and tested for their radical-scavenging activity. At the same time, the seed yield, oil and protein content and oil yield as well as fatty acid profile, were investigated for all the tested varieties in both environments, providing useful information about camelina and flaxseed yield potential under the climate conditions of Mediterranean region.

## 2. Results

### 2.1. Seed Yield and Qualitative Characteristics

**Figure 1:**
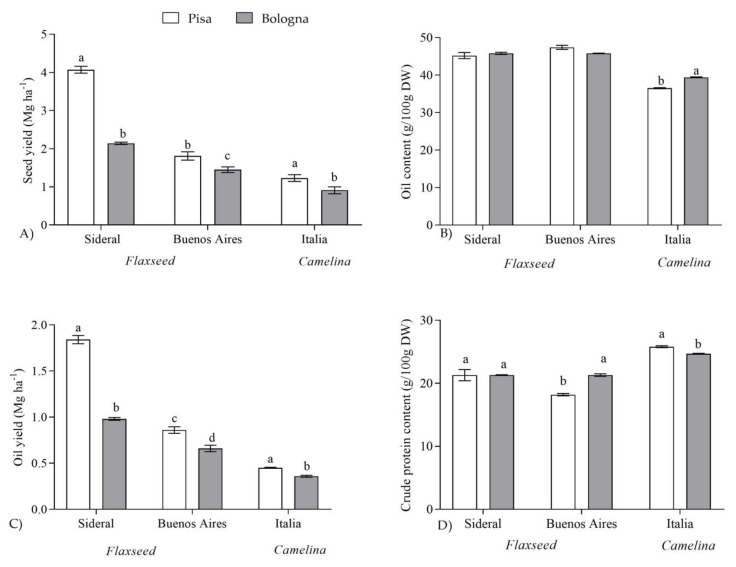
Seed yield (**A**), oil content (**B**), oil yield (**C**) and crude protein content (**D**) of the two oilseed crops (mean^†^ ± SD) grown in two cultivation sites (Pisa and Bologna). ^†^ Values are the means of four replicates. Means followed by different letters are significantly different according to LSD_0.05_ or Student’s *t*-test.

**Figure 2:**
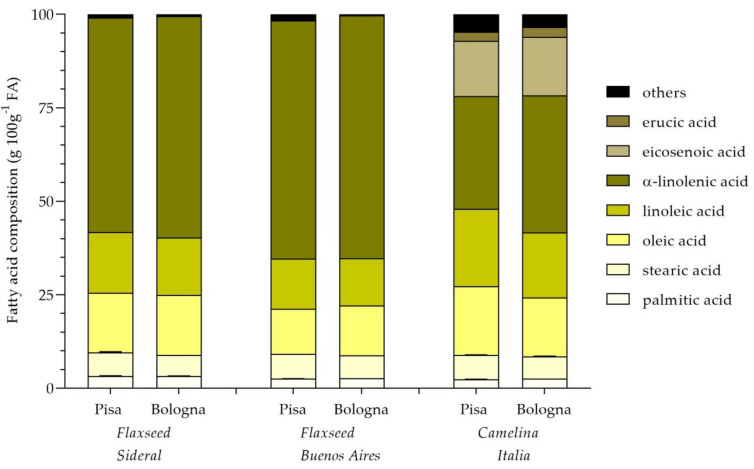
Fatty acid composition of oils of flaxseed (Sideral and Buenos Aires) and camelina (Italia) grown in two cultivation sites (Pisa and Bologna).

In Figure 1 and Figure 2, the main agronomic (seed and oil yield) and qualitative (seed oil and protein contents and fatty acid profile) traits, for all oilseed crops and sites were reported. Regarding flaxseed, the highest seed yield was obtained for Sideral in Pisa, while Buenos Aires in Bologna was characterized by the lowest crop yield. The same trend was observed for oil seed yield, with the highest value in Pisa for Sideral and the lowest one, once again, for Buenos Aires, grown in Bologna. On the contrary, no significant differences were found for oil content (%), indicating that the seed oil yield depended on seed yield rather than seed oil content. The seeds of Buenos Aires cultivated in Pisa were characterized by the lowest crude protein content (Figure 1).

Regarding camelina, the crop grown in Pisa showed the highest seed yield as well as the highest seed oil content, oil yield, and protein amount (Figure 1).

In Figure 2, the fatty acid composition of flaxseed and camelina seeds has been shown. Data highlighted that, for both flaxseed varieties, fatty acid composition did not vary depending on the cultivation site, while differences were observed between the two cultivars. In particular, Buenos Aires seeds were characterized by a lower content of linoleic acid and a higher content of α-linolenic one, in comparison with Sideral. On the contrary, for camelina, the cultivation site seemed to have a significant effect on the acidic composition, with higher contents of oleic and linoleic acids and a lower amount of α-linolenic acid in seeds obtained from the Pisa crop, compared with the fatty acid profile of camelina seed obtained in Bologna.

### 2.2. Phytochemical Screening and Anti-Radical Activity of Seed Meals 

In Table 1 and Table 2, the content of total phenols and flavonoids and anti-radical activity of flaxseed and camelina seed meals are shown. Regarding the phytochemical characteristics of flaxseed meal, ANOVA analysis showed no significant effect of variety and cultivation site on both the TPC (total phenolic content) and TFC (total flavonoids content) (Table 1).

A similar trend was also observed for anti-radical activity (Table 2), even if significant differences have been observed between varieties and sites for EC_50_ estimated by the DPPH assay. In particular, lower values, indicating a major anti-radical activity, were registered for meal obtained from Buenos Aires seeds and in general, for flaxseed meals deriving from the seeds produced in Pisa (Table 2). In camelina, the highest value of TPC was found in the defatted meal deriving from the seeds produced in Pisa (Table 1), while no differences between the two cultivation sites were observed for the flavonoid content. According to the phenol content, seed meals deriving from camelina produced in Pisa were characterized by the lowest anti-radical activity. The lower the EC_50_ value is, the higher the extract ability to scavenge radicals is, particularly peroxy radicals, which are the propagators of the autoxidation of lipid molecules and thereby break the free radical chain reaction (Table 2).

### 2.3. LC–PDA/UV–ESI–MS Profiles

#### 2.3.1. Lignan Content of Flaxseed Meal

**Figure 3:**
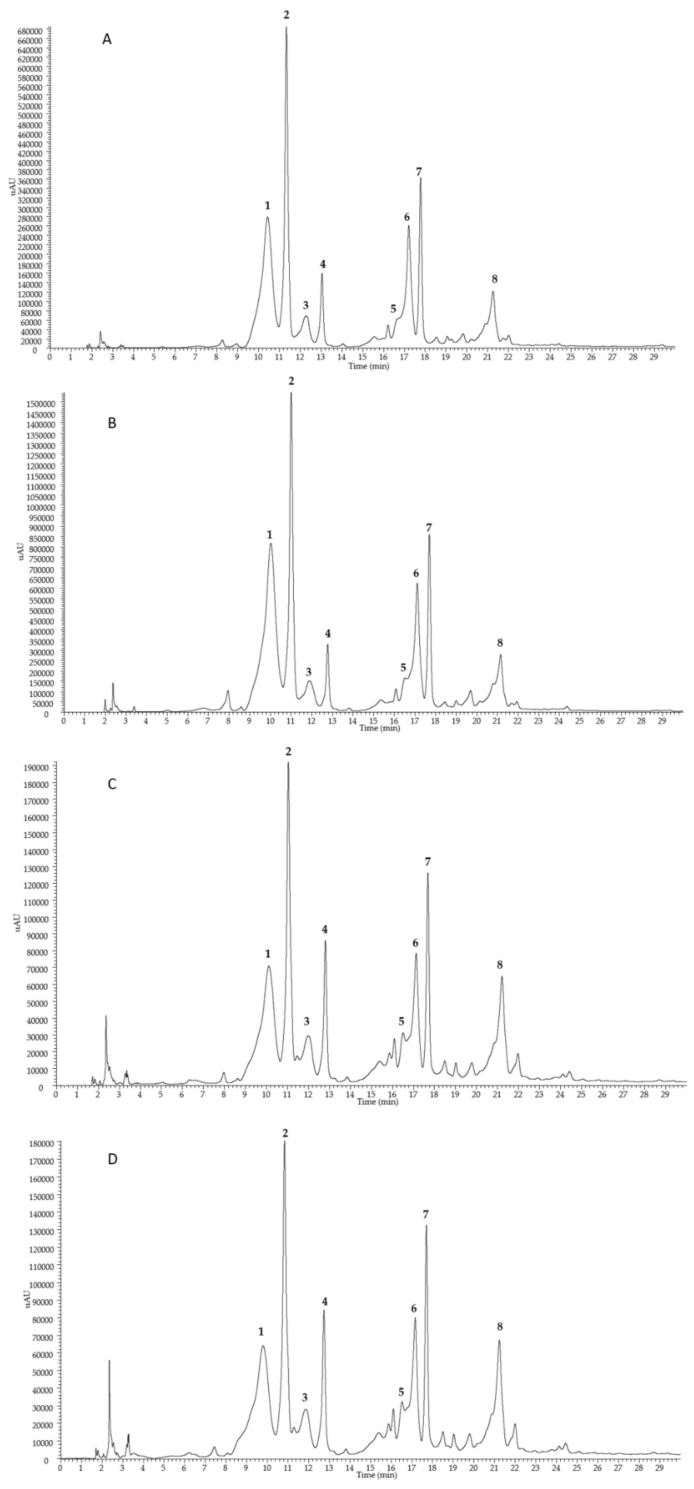
HPLC–PDA/UV profiles (detected at 280 nm) of secoisolariciresinol diglucoside (SDG) oligomers alkaline hydrolysates from the defatted seed meal extracts of flaxseed Sideral cultivated in Bologna (**A**) and Pisa (**B**), and Buenos Aires cultivated in Bologna (**C**) and Pisa (**D**). For peak data, see Table 3.

Flaxseed is known as a major source of lignan SDG, which is present in the form of high molecular oligomers due to ester bonds with 3-hydroxy-3-methylglutaric acid (HMGA) and glycosidic linkages with phenolic compounds, such as hydroxycinnamic acid derivatives and herbacetin diglucoside [14]. Both alkaline and acid hydrolysis of SDG oligomers is commonly used to analyze the lignan content of flaxseed [15]. In the present study, the chemical characterization of flaxseed lignans from the four analyzed extracts (defatted seed meal of flaxseed Sideral and Buenos Aires in the two cultivation sites Pisa and Bologna) was performed on the alkaline hydrolysates of SDG oligomers by the means of HPLC coupled to a PDA/UV detector and an electrospray ionization mass spectrometer (ESI–MS). The PDA/UV chromatograms (Figure 3) were acquired at 280 nm, which is the maximum absorption of SDG. All the extracts showed very similar profiles, with the presence of phenolic compounds due to the breaking of oligomer ester linkages. Indeed, the alkaline hydrolysis led to the formation of phenolic acid glucosides, such as *p*-coumaric acid glucoside (two isomeric forms, peaks **1** and **2**) and ferulic acid glucoside (two isomeric forms, peaks **3** and **4**), the flavonoid herbacetin diglucoside (peak **5**), the lignan SDG (two isomeric forms, peaks **6** and **7**), and ferulic acid (peak **8**), according to previous studies [15]. The tentative identification of all compounds was carried out comparing their elution order, ESI–MS/MS and PDA/UV data (Table 3) with those previously reported [16].

Masses of identified phenolics were detected in negative ion mode, originating deprotonated [M − H]^−^ molecules and except for compound **6**, formiate [M + HCOO]^−^ and acetate [M + CH_3_COO]^−^ adducts, leading to establish the molecular weight of detected substances. MS/MS of [M + CH_3_COO]^−^ ions for compounds **1**/**2** (*m/z* 385) and **3**/**4** (*m/z* 415) showed losses of a hexosyl moiety ([M−162]^−^) due to the cleavage of the *O*-sugar bond, generating aglycon portions attributable to *p*-coumaric acid and ferulic acid, respectively. Thus, compounds **1**/**2** and **3**/**4** were identified as two isomeric forms of *p*-coumaric acid glucoside and ferulic acid glucoside, respectively, that cannot be distinguished on the basis of UV and MS data. The alkaline hydrolysates showed also the presence of ferulic acid (**8**), with λ_max_ 237 and 323 nm and diagnostic product ions (*m/z* 178, 149, and 134) generated in the MS/MS of deprotonated molecule [M − H]^−^ at *m/z* 193. The full mass spectrum of compound **5** showed a deprotonated molecule [M − H]^−^ at *m/z* 625, while MS/MS displayed two diagnostic fragment ions at *m/z* 463 and 301 generated by the subsequent losses of two hexosyl moieties. Thus, compound **5** was identified as herbacetin diglucoside, a flavonol considered part of the lignan macromolecule [17]. Compounds **6** and **7** were identified as two isomeric forms of SDG, the most abundant lignan present in linseed. The MS/MS experiment for the acetate adduct [M + CH_3_COO]^−^ at *m/z* 745 provided a product ion at *m/z* 583, due to the loss of a hexosyl moiety, according to the presence of a glucosyl residue. A few minor peaks remained unidentified.

**Figure 4:**
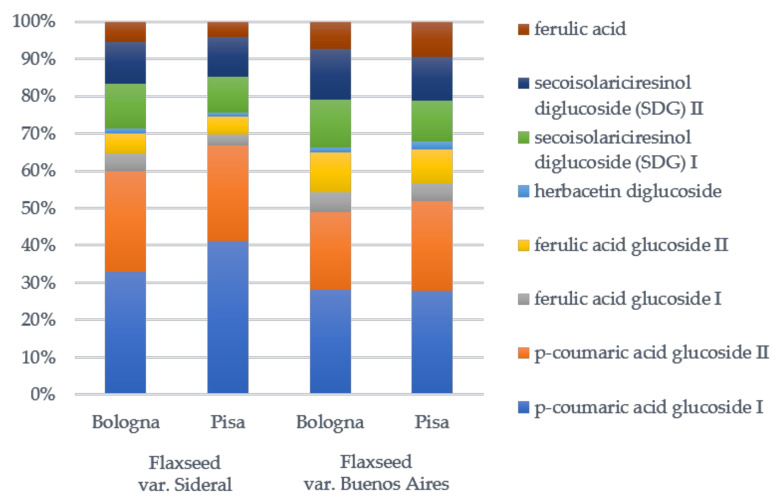
Percentage amount of identified phenols in secoisolariciresinol diglucoside (SDG) oligomers after the alkaline hydrolysis of flaxseed meal extracts from Sideral and Buenos Aires in two Italian cultivation sites, Pisa and Bologna.

The percentage composition of SDG oligomers in terms of detected phenols **1**–**8** after alkaline hydrolysis, calculated by integrating the peak areas at 280 nm in all camelina extracts, is shown in Figure 4. The most representative compound was *p*-coumaric acid glucoside (isomers **1** and **2**) with a percentage amount ranging from 48.8 to 60.0%, followed by SDG (isomers **6** and **7**; 20.2–26.4%), ferulic acid glucoside (isomers **3** and **4**, 7.6–16.0%), ferulic acid (**8**. 4.2–9.4%), and herbacetin diglucoside (**5**. 1.0–2.1%). Based on these results, meal extract from flaxseed Sideral cultivated in Pisa was the richest in *p*-coumaric acid glucoside, while meal extract from flaxseed Buenos Aires cultivated in Bologna was the richest in SDG and ferulic acid glucoside.

#### 2.3.2. Glucosinolate and Phenol Contents of Camelina Meal

**Figure 5:**
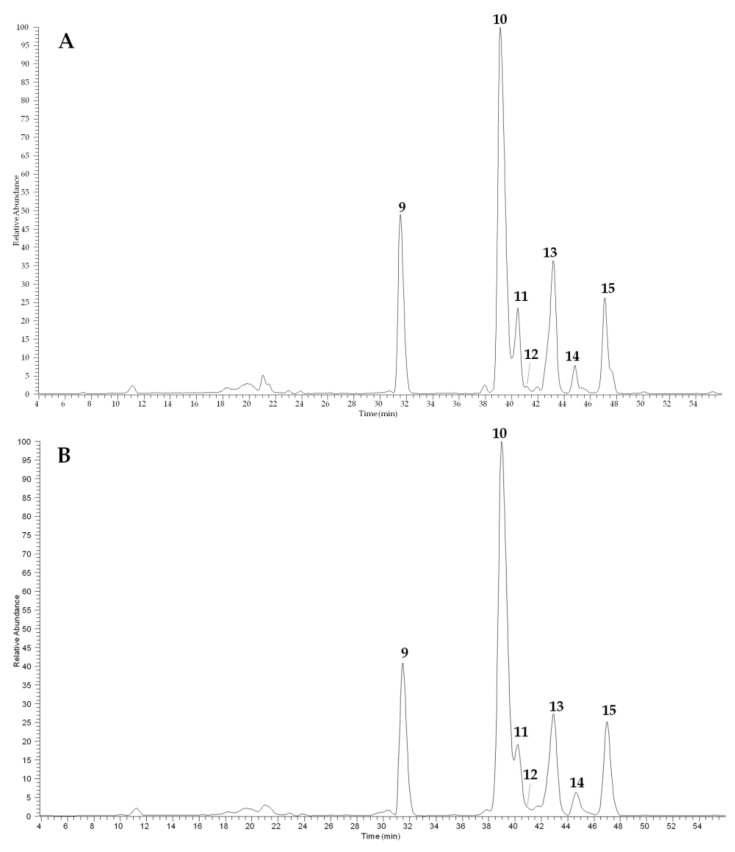
HPLC–ESI–MS profiles (registered in negative ion mode) of camelina meal extracts cultivated in Pisa (**A**) and Bologna (**B**). For peak data, see Table 4.

The LC–ESI–MS analyses of camelina meal extracts (Figure 5), registered in negative ion mode, showed the presence of two major classes of compounds, represented by glucosinolates (peaks **9**, **10**, and **15**) and flavonoids (peaks **11**–**13**). The compounds were tentatively identified on the basis of spectral data. The MS/MS of all glucosinolates ([M − H]^−^ at *m/z* 506, 520, and 534) showed diagnostic fragments due to the losses of a SO_2_ molecule and were identified as glucoarabin (**9**), glucocamelinin (**10**), and 11-(methylsulfinyl)undecylglucosinolate (**15**), in agreement with previous studies [18,19]. The three detected flavonoids, characterized by two strong UV absorptions at 256–258 and 350–356 nm were all in the form of glycosides characterized by the presence of the same aglycone identified as quercetin due to the diagnostic fragment at *m/z* 301 in the MS/MS. Compound **11** ([M − H]^−^ at *m/z* 741) was a triglycoside as deduced by its fragmentation pathway ([M − H − 132 − 146 − 162]^−^) and was identified as quercetin 2”-*O*-apiosyl-3-*O*-rutinoside, previously isolated from camelina by Quéro et al. [18]. Both compounds **12** ([M − H]^−^ at *m/z* 595) and **13** ([M − H]^−^ at *m/z* 609) contain a disaccharide chain constituted by pentose–hexose and deoxyhexose–hexose, respectively. Thus, compound **13** was identified as quercetin 3-*O*-rutinoside (rutin) [19], while compound **12** is probably a quercetin apiosyl-glucoside in which the exact position of sugars cannot be determined only on the basis of MS/MS data; based on similar components previously found in camelina seeds, it can be assumed that compound **12** is quercetin 2”-*O*-apiosyl-3-*O*-glucoside. Finally, compound **14** ([M − H]^−^ at *m/z* 623) remained not completely identified, but it could be a synapoil derivative as can be deduced by the presence of fragment [M − H − 206]^−^ at *m/z* 417.

The LC–MS quantitative analyses (Table 5) showed that among glucosinolates glucocamelinin was the most representative in both camelina meal extracts, while rutin was the most abundant among flavonoids. Furthermore, meal extract from camelina cultivated in Bologna showed the highest content in term of glucosinolates [18.6 ± 0.3 vs. 14.5 ± 0.5 mg/g dry weight (DW)], whereas meal extract from camelina cultivated in Pisa showed the highest content in terms of flavonol glycosides (4.8 ± 0.08 vs. 4.5 ± 0.06 mg/g DW).

## 3. Discussion 

The obtained results showed that both flaxseed and camelina were able to reach satisfactory yields in both environments, even if clear effects of environment and variety were observed, with the highest productive performance reached by Sideral and Italia grown in Pisa. The obtained results are consistent with those reported in the literature for different environments and cultivars [4,20,21,22]. The analysis of the chemical composition of flax seeds showed that oil contents were not significantly different under the influence of variety or environmental conditions, with average values of 46%. This content was higher in comparison with those reported in previous studies [23,24] in which flaxseed oil content ranged between 34 and 45%, depending on the geographical area, genotype and environmental conditions. Flaxseeds were characterized by a very stable proportion of polyunsaturated fatty acids, in both varieties and environments, with α-linolenic acid as the most abundant fatty acid. It is known that this fatty acid is characterized by beneficial effects on the prevention of several diseases, such as cardiovascular diseases, hypercholesterolemia, chronic kidney diseases, atherosclerosis, and neurological disorders [25,26]. In the present study, the content of α-linolenic acid in both flaxseed varieties, ranging from 57 to 65%, consistent and sometimes higher than that reported in literature (from 50 to 59%, depending on genotype and environment) [27,28].

In camelina, oil content and fatty acid profiles were dependent on the environment, with higher oil and oleic acid contents in Pisa samples, compared to the Bologna ones. On the contrary, the seed oil of camelina grown in Bologna showed an increased content of eicosenoic acid and α-linolenic one. These differences could be due to the highest temperatures experienced during flowering and seed filling by the crop cultivated in Pisa, for which a spring sowing was performed. It is known that elevated temperatures during flowering and seed ripening determine a rise in oleic acid since temperature can interfere with the activity of the enzymes involved in the biosynthesis of fatty acids [29]. Interestingly, eicosenoic acid can be a valuable source of medium chain fatty acids for the bio-based industry, which nowadays are not produced in Europe as they are totally derived from palm and coconut oils [30]. Nonetheless, the oil content fell in the range typically reported for this oilseed crop [21,30]. Finally, as a general observation, the crude protein content of both types of oilseeds was negatively correlated with the oil content confirming previous findings [5,31].

The evaluation of the total phenols and flavonoids and their related antioxidant activities showed that both types of meal were characterized by interesting levels of these beneficial substances, regardless of the variety and growing environment, except for that given for total phenolic content and the anti-radical activity of camelina meals. In this case, in fact, higher phenols and stronger anti-radical activity, measured by DPPH, were observed for defatted meal deriving from camelina grown in Pisa, in comparison with camelina meal obtained from Bologna. In the literature, regarding total phenols and flavonoids in flaxseed and camelina cakes and/or meals, lower and higher values are reported, in comparison with our findings, depending on the variety, extraction method, and growing conditions [32,33,34,35]. For example, Teh and Birch [32] found, in defatted flaxseed cake, levels of total phenols and total flavonoids in the range of 474–807 mg GAE/100 g FW (FW = fresh weight) and 5.6–15.6 mg luteolin equivalents (LUE)/100 g FW, respectively, depending on the extraction method (ultrasonic and conventional method), solvent volume and extraction temperature. In the defatted camelina meal, Rahman et al. [34] observed a mean total phenolic content of 11.69 ± 0.44 mg GAE/g DW with a total flavonoid content equal to 6.81 ± 0.68 mg CAE/g DW.

Phenolic compounds are recognized as important food metabolites able to prevent several pathologies, such as cardiovascular and neurodegenerative diseases and cancer [36,37]. Polyphenols exhibit, in fact, interesting antioxidant activity thanks to their ability to transfer a hydrogen atom or an electron, acting as a reducing agent, as well as by the possible chelation of metal ions and the inhibition of the activity of oxidases [38]. The antioxidant activity of the hydroalcoholic extracts of the two kinds of meals tested in the present study was in line with the results on phenol and flavonoid concentration. In fact, a lower value of EC_50_ (for both DPPH and ABTS assays) were revealed for camelina meal, suggesting a positive correlation with the higher phenols and flavonoids detected in this kind of meal. The lower the EC_50_ value is, in fact, the higher the ability of the extract to scavenge radicals is. Previous reports underlined that camelina and flaxseed meals, after solvent oil extraction, contain good amounts not only of phytochemicals but also crude proteins (32–45%, with the presence of important essential amino acids, such as lysine, methionine and cysteine), insoluble fiber, carbohydrates and minerals [34,39,40,41], which make these meals good candidates for food and feed applications. 

As expected, all flaxseed meal extracts were found to be a good source of SDG, herbacetin diglucoside, and hydroxycinnamic acid glucosides (ferulic and *p*-coumaric acid glucosides), herein characterized by HPLC–UV–MS analyses of alkaline hydrolysate, since they are accumulated in flaxseed seeds in form of oligomers [15]. Although the chemical compositions of the four extracts showed the same profiles in terms of constituents and their relative abundance, Buenos Aires flaxseed’s meal cultivated in Bologna resulted, even if with small distances from others, the major source of SDG, a lignan whose potential health benefits are under investigation in many recent studies [8]. SDG, one of the most representative monomeric constituents of the lignan macromolecule, is reported to have many biological activities such as antioxidant and anti-inflammatory properties, playing a role in the prevention against chronic diseases, such as cardiovascular events and metabolic syndrome [8].

In agreement with previous investigations [18], three major glucosinolates were found in camelina meals, showing glucocamelinin as the most representative (about 64 and 66% of the total glucosinolate composition in the varieties growing in Pisa and Bologna, respectively). Long-chain glucosinolates predominate in camelina compared with short-chain glucosinolates that comprise the majority of glucosinolates in canola meal. Previous studies evidenced that glucosinolate content in camelina seeds is dependent on the geographic origin of seeds, as well as climatic factors and soil conditions [9]. A large variation was also observed between different cultivars [42]. In a recent work, Russo and Reggiani [43] reported an amount of total glucosinolates ranging from 19.6 to 40.3 mmol/kg DW in camelina meal from 47 accessions, with an average of 30.3 mmol/kg DW. Compared to these data, the amount of total glucosinolates in camelina meal obtained in the present work from the two Italian cultivation sites were moderately high, with camelina from Bologna richer (35.7 mmol/kg DW) than camelina from Pisa (27.9 mmol/kg DW), probably due to the different environmental conditions. In addition to glucosinolates, both camelina extracts were shown to contain phenolic constituents in quite a similar amount, with camelina from Pisa slightly above camelina from Bologna. Flavonoids, mainly kaempferol and quercetin derivatives, were previously reported in camelina seeds [19] and their profile compared to that of camelina meal [34,35]. The LC–MS analyses of camelina meal extracts from Pisa and Bologna showed that both samples contained three major quercetin glycosides, with quercetin 3-*O*-rutinoside the most representative, followed by quercetin 2”-*O*-apiosyl-3-*O*-rutinoside, herein more expressed than previous studies. The presence of glucosinolates and flavonoids is important for defining the nutraceutical value of camelina meals. Glucosinolates, secondary metabolites typical of the Brassicaceae family, have received great attention for their potential benefits in the prevention of carcinogenesis as well as cardiovascular and neurological diseases [44,45,46]. In a recent preliminary study, glucocamelinin and glucoarabin from defatted seed meal showed an ability to upregulate the phase II detoxification enzyme quinone reductase (NQO1) [18]. Similarly, rutin, a common flavonol glycoside found in a large number of plant species, is known for its antioxidant activity and its role in the treatment and prevention of various diseases [47].

## 4. Materials and Methods

### 4.1. Reagents and Standards

Methanol, formic acid and acetic acid for HPLC–MS analyses, and all analytical grade solvents and reagents were purchased from VWR (Milano, Italy). Water HPLC grade (18 mΩ) was prepared by Mill-Ω purification system (Millipore Co., Bedford, MA, USA). Folin–Ciocalteu reagent was purchased from Merck (Darmstadt, Germany). ABTS and DPPH were purchased from Sigma Aldrich (St. Louis, MO, USA). Rutin (purity ≥ 99%) and glucoraphanin (purity ≥ 98%) were purchased from Extrasynthese (Genay, France).

### 4.2. Experimental Conditions and Plant Material

Field plot experiments were carried out during the 2013–2014 growing season at the Centre for Agro-Environmental Research “Enrico Avanzi” of the University of Pisa located in San Piero a Grado (Pisa, central Italy, 43°40′ N; 10°19′ E, 1 m above sea level) and at the CREA (Council for Agricultural Research and Economics) experimental farm in Budrio (Bologna, northern Italy, 44°32′ N; 11°29′ E, 28 m above sea level), by adopting a randomized block design with four replicates (plots size of 6.5 m × 3.0 m) for each species and/or cultivars. Both sites were characterized by flat land with alluvial deep loam soils. In Pisa, the soil was a typic Xerofluvent, representative of the lower Arno river plain, characterized by a low level of organic matter (1.7%), and a medium content of available phosphorous (12.0 mg/kg) and total nitrogen (1.1 g/kg), with a moderately alkaline reaction (pH 8.2) and slightly calcareous (total CaCO_3_ 3.1%). In Bologna, the soil was moderately alkaline (pH 8.1), characterized by good contents of organic matter (2.1%) and total nitrogen (1.4 g/kg), very good level of available P (33.3 mg/kg), and moderately calcareous (total CaCO_3_ 10.3%). In both environments, the two flaxseed varieties used in the experiment were Sideral and Buenos Aires. Sideral is a variety registered in the EC (European Commission) common catalogue of varieties and commercially available (Semfor s.r.l., Verona, Italy), characterized by high resistance to cold and lodging and early ripening with blue-violet flowers and brown colored seeds [5]. Buenos Aires belongs to the germplasm collection of CREA-CI (Bologna, Italy), and it is characterized by a low cold resistance and an early ripening, with white flowers and yellow-colored seeds. Regarding camelina, the cultivar Italia, belonging to CREA-CI germplasm collection, was used [48]. The previous crop in both locations was durum wheat (*Triticum turgidum* L. subsp. *durum* (Desf.) Husn.), assuming a rotation with cereals. All flaxseed crops were sown during the fall, from mid- to the end of October, after the cereal crop harvest. Camelina was analyzed as a separate experiment comparing a winter crop in Bologna (sown in mid-October 2013) with a spring crop in Pisa (sown in mid-March 2014). Both crops have been harvested from the beginning to the end of June, at full seed maturity.

### 4.3. Agronomic Evaluations 

At full seed maturity, four randomized sample areas of 2 m^2^ were collected within each experimental plot for each crop and in each environment to assess harvestable crop yield. The plants were manually cut and gathered and then threshed by a fixed machine, using sieves suitable for small seeds, and evaluated for their moisture and seed yield.

### 4.4. Seed Processing and Analysis

Seed moisture was determined by oven-drying the seeds at 40 °C until constant weight for dry weight determination and the moisture content was calculated as the difference between the seed weight before and after the treatment. Oil was extracted by Buchi E-816 ECE (Soxhlet-like extractor) for 210 min, with hexane and *trans*-methylated in 2N KOH methanol solution [49]. Fatty acid profile was evaluated by a gas chromatography equipped with a flame ionization detector (Carlo Erba HRGC 5300 MEGA SERIES) and a capillary column Restek RT × 2330 (30 m × 0.25 mm × 0.2 μm), following the internal normalization method (ISO 12966–4:2015). The crude protein content was expressed as the percentage of dry matter and calculated from nitrogen using the conventional factor of 6.25. The obtained meals were dried at room temperature, vacuum-sealed and then stored away from light and heat, until the subsequent analysis.

### 4.5. Extraction of Bioactive Compounds

Flaxseed and camelina meals (0.25 g) were extracted with 5 mL of methanol–water (80% v/v) and sonicated for 30 min at room temperature. After centrifugation, the supernatant was filtered through a sterile 0.45 μm Minisart Syringe Filter and the resulting extracts were stored at −20 °C before use for up to a week.

### 4.6. Analysis of Total Phenols and Flavonoids

Total phenols were determined using the Folin–Ciocalteu method according to Singleton et al. [50]. The absorbance of the blue complex formation was determined at 765 nm by UV–vis spectrophotometer (Varian Cary 1E, Palo Alto, CA, USA). The results were expressed as mg gallic acid equivalent (GAE) per gram of seed meal on dry basis. Total flavonoids were determined using the method described by Jia et al. [51] measuring the absorbance of the pink complex at 510 nm using a UV–vis spectrophotometer (Varian Cary 1E, Palo Alto, CA, USA). The results were expressed as mg catechin equivalent (CAE) per gram of seed meal on dry basis. Measurements were replicated three times for each sample.

### 4.7. HPLC–PDA/UV–ESI–MS/MS Analyses of Camelina and Flaxseed Meal Extracts

#### 4.7.1. Alkaline Hydrolysis of Flaxseed Oligomers

For the hydrolysis of the SDG oligomers, the four dried flaxseed extracts were dissolved in methanol (100 mg/mL) and mixed to an equal volume of 2M NaOH solution. The alkaline hydrolysis was carried out for 2 h at room temperature, then stopped by the addition of HCl 36% (1.2 M final concentration) [52]. The samples were successively centrifugated. Each supernatant was finally subjected to HPLC analysis at a concentration of 2.0 mg/mL.

#### 4.7.2. HPLC–UV–MS Analyses

After alkaline hydrolysis, the chemical content of each flaxseed meal extract, together with camelina meal extracts, was analyzed by HPLC–PDA/UV–ESI–MS/MS technique. The LC–PDA/UV ESI–MS system was composed by a Surveyor LC pump, a Surveyor autosampler, coupled with a Surveyor PDA detector, and a LCQ Advantage ion trap mass spectrometer (ThermoFinnigan, San Jose, CA, USA) equipped with Xcalibur 3.1 software.

Analyses were performed using a 4.6 × 250 mm, 4 µm, Synergi Fusion-RP column (Phenomenex, Bologna, Italy). The eluent was a mixture of methanol (solvent A) and a 0.1% v/v aqueous solution of acetic acid (solvent B). For flaxseed meal extract analysis, a linear gradient of increasing 5 to 35% A was developed within 30 min, while for camelina meal extract, a linear gradient of increasing 5 to 60% A was used within 55 min. The column was successively washed with methanol an equilibrated with 5% A for 10 min. 

Elutions were performed at a flow rate of 0.8 mL/min with a splitting system of 2:8 to MS detector (160 μL/min) and PDA detector (640 μL/min), respectively [53]. The volume of the injected methanol solutions was 20 μL. Analyses were performed with an ESI interface in the negative ion mode. The ionization conditions were optimized and the parameters used were as follows: capillary temperature, 270 °C; capillary voltage, −16.0 V; tube lens offset, −5 V; sheath gas flow rate, 60.00 arbitrary units; auxiliary gas flow rate, 3.00 arbitrary units; spray voltage, 4.50 kV; scan range of *m/z* 150–1500. N_2_ was used as the sheath and auxiliary gas. PDA data were recorded within the 200–600 nm range, with the UV preferential channel as the detection wavelength of 280 nm.

For quantitative analyses of glucosinolates and flavonoids in camelina seed cake extracts, calibration curves were constructed by using glucoraphanin (concentration range 0.06–0.5 mg/mL) and rutin (concentration range 0.01–0.25 mg/mL), as external standards, respectively. Standard methanol solutions at different concentrations were prepared by serial dilution from stock solution (1 mg/mL), then analyzed by triplicate injections, and finally used with respect to the area obtained from the integration of the MS base peak [M − H]^−^ of each standard to generate a calibration curve. The relations between variables were analyzed by using a linear simple correlation (*R*^2^ = 0.9829 for rutin and 0.9837 for glucoraphanin). The phenol amounts were obtained by using a Microsoft^®^ Office Excel (Redmond, WA, USA) and finally expressed as mg/g of dried cakes.

### 4.8. Free Radical-Scavenging Assay

The free radical-scavenging activity was evaluated by the DPPH (1,1-diphenyl-2-picryl-hydrazil radical) and ABTS free radical assay according to the method described by Brand-Williams et al. [54] and spectrophotometrically estimating the solution discoloration at 515 and 734 nm, respectively. The concentration required to obtain a 50% antioxidant effect (EC_50_) was evaluated as the concentration of extract (mg of defatted seed meal × mL^−1^ of extraction solvent) causing the 50% inhibition of the initial color production. A series of dilutions in 80% methanol was prepared for each extract and standard. An Infinite M200 PRO microplate reader was used for spectrophotometric assays. Trolox and butylated hydroxyanisole (BHA) were used as the reference standards. Measurements were replicated three times for each sample.

### 4.9. Statistical Analyses

All the agronomic and phytochemical variables were subjected to the analysis of variance (ANOVA) using the statistical software CO-STAT Cohort, 2002 (CoHort Software, Monterey, CA, USA). A factorial design with variety (V) and cultivation site (S) as main treatments was used for flaxseed deriving data. Means were separated on the basis of least significance difference (LSD) post-hoc test only when the ANOVA *F*-test per treatment was significant at ≤ 0.05 probability level. For the camelina data, a Student’s *t*-test analysis was performed in order to estimate the effect of a cultivation site.

## 5. Conclusions

In summary, this study pointed out the interesting functional properties of flaxseed and camelina meals, thanks to the abundant presence of antioxidants and phytochemicals, which represent high-value components of these important plant-derived products. In particular, all the examined flaxseed meals resulted as rich in phenol content, as deduced from LC–UV–MS analyses of each extract after alkaline hydrolysis of SDG oligomers. On the other hand, camelina defatted meals, showed comparable content of glucosinolates and flavonols glycosides quercetin derivatives, with glucocamelinin and rutin the most representative, respectively.

Thus, flaxseed and camelina meals investigated in the present work could be considered in further biological studies as potential dietary sources of health-beneficial compounds, able to play an important role in the reduction in the incidence of non-communicable diseases, including obesity, diabetes, cancer, and other chronic conditions. Thanks to their interesting chemical composition, their edible defatted meals can be used in human consumption as processed ingredients and/or as a source of antioxidants, and incorporated, for example, in bakery, infant products, and multipurpose supplements. At the same time, taking into account the increasing environmental issues, the use of these co-products and the recovering/recapturing of valuable components can be a sustainable tool for reducing waste disposal and developing new environmental-friendly functional foods and/or ingredients.

## Figures and Tables

**Table 1:** Total phenols and total flavonoids (mean^†^ ± SD) in the defatted seed meal of flaxseed (Sideral and Buenos Aires) and camelina (Italia) from two cultivation sites (Pisa and Bologna).

Crop/Variety	Total Phenolic Content (mg GAE/gDW)			Total Flavonoids (mg CAE/gDW)		
	Pisa	Bologna	*Mean Variety*	Pisa	Bologna	*Mean Variety*
Flaxseed Sideral	2.57 ± 0.28	2.74 ± 0.39	2.66	1.27 ± 0.05	1.36 ± 0.19	1.32
Flaxseed B.Aires	2.86 ± 0.15	2.74 ± 0.17	2.80	1.16 ± 0.23	1.23 ± 0.16	1.20
*Mean Site*	2.71	2.74		1.22	1.30	
*Significance*	*Variety (V) = n.s.*			*Variety (V) = n.s.*		
	*Site (S) = n.s.*			*Site (S) = n.s.*		
	*VxS = n.s.*			*VxS = n.s.*		
Camelina Italia	7.00 ± 0.18 a	6.26 ± 0.28 b	6.63	6.15 ± 0.79	5.48 ± 0.45	5.82
*Significance*	Site (S) = **			*Site (S) = n.s.*		

^†^ Values are the means of four replicates. Significance of variability factors according to F-test is reported as follows: n.s., not significant; **, significant at *p* ≤ 0.01 level. For camelina’s total phenolic content, means followed by different letters are significantly different at *p* ≤ 0.05 based on the Student’s *t*-test. CAE: catechin equivalent; DW: dry weight; GAE: gallic acid equivalent; SD: standard deviation.

**Table 2:** Anti-radical activity (expressed as EC_50_ and evaluated by DPPH and ABTS assays) in the defatted seed meal of flaxseed (Sideral and Buenos Aires) and camelina (Italia) in two cultivation sites (Pisa and Bologna). EC_50_ values of Trolox and BHA (butylated hydroxyanisole) were also shown as reference. Data are expressed as the mean^†^ ± SD.

Crop/Variety	EC_50_ DPPH (mg mL^−1^)			EC_50_ ABTS (mg mL^−1^)		
	Pisa	Bologna	*Mean Variety*	Pisa	Bologna	*Mean Variety*
Flaxseed Sideral	3.60 ± 0.20	4.30 ± 0.30	3.95 A	3.10 ± 0.30	2.80 ± 0.20	2.95
Flaxseed B.Aires	3.10 ± 0.20	3.80 ± 0.20	3.45 B	3.00 ± 0.30	3.20 ± 0.30	3.10
*Mean Site*	3.35 B	4.05 A		3.05	3.00	
*Significance*	Variety (V) = **			*Variety (V) = n.s.*		
	Site (S) = ***			*Site (S) = n.s.*		
	*VxS = n.s.*			*VxS = n.s.*		
Camelina Italia	1.50 ± 0.10 b	1.90 ± 0.10 a	1.70	2.10 ± 0.30 b	2.90 ± 0.30 a	2.50
*Significance*	Site (S) = **			Site (S) = **		
*Trolox*	0.052 ± 0.001			0.046 ± 0.001		
*BHA*	0.031 ± 0.001			0.029 ± 0.001		

^†^ Values are the means of four replicates. SD: standard deviation. Significance of variability factors according to *F*-test is reported as follows: n.s., not significant; **, significant at *p* ≤ 0.01 level; ***, significant at *p* < 0.001 level. Means followed by different letters are statistically different at *p* ≤ 0.05 based on an LSD test or Student’s *t*-test.

**Table 3:** Spectral (UV and ESI–MS/MS) and chromatographic data (*t*_R_, retention time) of compounds **1**–**8**, detected in all defatted seed meal extracts of flaxseed after the alkaline hydrolysis of secoisolariciresinol diglucoside (SDG) oligomers.

Peak ^a^	Compound	*t*_R_ (min)	[M − H]^−^	[M + CH_3_COO]^−^	[M + HCOO]^−^	MS/MS Ions (*m/z*) ^b^	λ_max_ (nm)
	*Phenolic acids*						
**1/2**	*p*-Coumaric acid glucoside	10.3, 11.2	325	385	371	325, 163	228, 295
**3/4**	Ferulic acid glucoside	12.1, 13.0	355	415	401	355, 193	235, 290
**8**	Ferulic acid	21.2	193	253	239	178, 149, 134	237, 323
	*Flavonoids*						
**5**	Herbacetin diglucoside	16.2	625	─	─	463, 301	235, 284, 323
	*Lignans*						
**6–7**	Secoisolariciresinol diglucoside (SDG)	17.1, 17.6	685	745	731	685, 583	232, 280

^a^ Compound numbers correspond to peak numbers in Figure 3. ^b^ For peaks **1**–**4**, **6**, and **7** MS/MS data were obtained by the fragmentation of the [M + CH_3_COO]^−^ precursor ions, whereas for peaks **5** and **8,** data were obtained by the fragmentation of the deprotonated molecule [M − H]^−^.

**Table 4:** Spectral (UV and ESI–MS/MS) and chromatographic data (*t*_R_, retention time) of compounds **9**–**15**, detected in camelina meal extracts.

Peak ^a^	Compound	*t*_R_ (min)	[M − H]^−^	MS/MS ions (*m/z*)	λ_max_ (nm)
	*Glucosinolates*				
**9**	Glucoarabin (9-(methylsulfinyl)nonylglucosinolate)	31.4	506	491, 442, 248	240
**10**	Glucocamelinin (10-(methylsulfinyl)decylglucosinolate)	38.9	520	505, 456, 262	239
**15**	11-(methylsulfinyl)undecylglucosinolate	47.1	534	519, 470	256
	*Flavonol glycosides*				
**11**	Quercetin 2”-*O*-apiosyl-3-*O*-rutinoside	40.2	741	609, 300, 301	256, 354
**12**	Quercetin *O*-apiosyl-glucoside	40.9	595	463, 300, 301	258, 350
**13**	Quercetin 3-*O*-rutinoside (rutin)	42.9	609	463, 301	257, 356
	*Other compound*				
**14**	Synapoil derivative	44.6	623	417, 399, 209	249, 328

^a^ Compound numbers correspond with peak numbers in Figure 5.

**Table 5:** Quantitative amount (mg/g ± SD DW) of constituents found in meal extracts from *Camelina sativa* Italia in two cultivation sites, Pisa and Bologna.

	Peak n. (in Figure 4)	Pisa	Bologna
*Glucosinolates*			
Glucoarabin	**9**	3.4 ± 0.04	3.9 ± 0.08
Glucocamelinin	**10**	9.3 ± 0.4	12.3 ± 0.2
11-(methylsulfinyl)undecylglucosinolate	**15**	1.8 ± 0.06	2.4 ± 0.04
Total		14.5 ± 0.5	18.6 ± 0.3
*Flavonol glycosides*			
Quercetin 2”-*O*-apiosyl-3-*O*-rutinoside	**11**	1.8 ± 0.03	1.7 ± 0.02
Quercetin apiosyl-glucoside	**12**	0.14 ± 0.004	0.21 ± 0.005
Quercetin 3-*O*-rutinoside	**13**	2.9 ± 0.05	2.6 ± 0.04
Total		4.8 ± 0.08	4.5 ± 0.06

DW: dry weight; SD: standard deviation.

## Data Availability

Data is contained within the article.
